# Machine-Learned Force Fields for Lattice Dynamics at Coupled-Cluster Level Accuracy

**DOI:** 10.1021/acs.jctc.6c00854

**Published:** 2026-07-01

**Authors:** Sita Schönbauer, Johanna P. Carbone, Fredrik Eriksson, Florian Libisch, Andreas Grüneis

**Affiliations:** † Institute of Theoretical Physics, Technical University of Vienna, Wiedner Hauptstraße 8−10, 1040 Vienna, Austria; ‡ Faculty of Physics and Center for Computational Materials Science, University of Vienna, Kolingasse 14-16, 1090 Vienna, Austria

## Abstract

We investigate machine-learned force fields (MLFFs) trained on approximate density functional theory (DFT) and coupled cluster (CC) level potential energy surfaces for the carbon diamond and lithium hydride solids. We assess the accuracy and precision of the MLFFs by calculating phonon dispersions and vibrational densities of states (VDOS) that are compared to experimental and reference ab initio results. To overcome limitations from long-range effects and the lack of atomic forces in the CC training data, a delta-learning approach based on the difference between CC and DFT results, as well as a charge-aware MLFF approach, is explored. Compared to DFT, MLFFs trained on CC theory yield higher vibrational frequencies for optical modes, agreeing better with the experiment. Furthermore, the MLFFs are used to estimate anharmonic effects on the VDOS of lithium hydride at the level of the CC theory.

## Graphical abstract

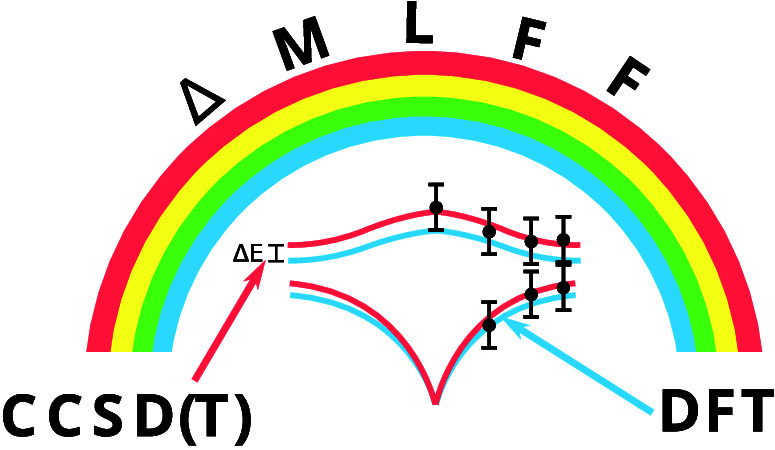

## 1 Introduction

Machine learning (ML) methods have become an invaluable asset in determining precise atomic force fields without having to explicitly keep track of the electronic problem.^1–7^ This allows for increases in system size and, in the case of Molecular Dynamics (MDs), longer simulation times at a comparatively negligible computational cost. However, the predictive accuracy of ML models is inherently limited by the quality of the training data used to generate them.

So far, most ML studies in the field of materials science are limited to data generated using approximate exchange and correlation density functionals. More accurate and systematically improvable Wave Function Theory (WFT) based methods such as the “gold standard” in quantum chemistry, Coupled Cluster (CC) theory at the level of single, double, and perturbative triple particle-hole excitations (CCSD­(T)), are computationally significantly more expensive, which severely limits the size of accessible training data.^8,9^ Consequently, these methods have been used mainly to generate training data for molecules.^10^ Recently, several studies have expanded their scope in the context of ML to calculate molecular adsorption energies in a zeolite structure and the properties of water.^11–13^ In this work, we investigate the extension of ML approaches to study the lattice dynamics of solids from training data generated by periodic CCSD­(T) theory.

Phonons play a fundamental role in determining various material properties, including heat capacity and electron–phonon coupling, which is closely related to the emergence of exotic states of matter such as superconductivity^14,15^ and quantum paraelectricity.^16,17^ Therefore, efficient and accurate computational methods for simulating phonons are essential for designing quantum materials with tailored and enhanced properties. In this context, DFT is persuasive with its great cost–accuracy trade-off for many materials. However, currently available density functionals sometimes fail to achieve the required levels of accuracy. A specific example is optical phonon frequencies in diamond, which are underestimated by commonly used approximate density functionals based on the generalized gradient approximation.^18^ Yet, highly accurate knowledge of the relationship between phonon frequency and pressure is needed for diamond anvil cell experiments.^18,19^

Training data containing energies and atomic forces substantially improves the trade-off between precision of the MLFF and the required training data size. However, there exist ab initio methods for which the calculation of atomic forces is difficult, because it requires the implementation of many terms, which might introduce excessive usage of memory. Therefore, it is also desirable to investigate approaches that are based on training data that exclude atomic forces. Currently, Δ- and transfer-learning approaches provide such an avenue,^10,20–24^ and there have also been promising results getting to CCSD­(T) accuracy without forces.^11^ Here, we restrict our investigation to a single Δ-learning technique for periodic CCSD­(T) theory because a larger number of different ML approaches would be beyond the scope of this work. Moreover, the number of systems studied is mostly limited by the relatively large computational cost of CCSD­(T) calculations.

Many successful approaches for MLFFs are described in literature. In particular, neural networks and regression models have become very popular in materials science during the last decades.^25,26^ We employ an equivariant message passing neural network referred to as MACE.^27,28^ The MACE implementation requires few input parameters, enabling a straightforward application to training data generated by periodic CC theory. Additionally, QNEP^29–31^ is used to bridge the gap to obtain long-range forces in the ionic LiH crystal; however, it is only trained at a DFT level used as the base for the Δ-learning approach.

The main goals of the present work are to (i) further assess the potential of Δ-learning approaches for periodic CCSD­(T) calculations, (ii) contribute to directing future developments in periodic CC theory needed to take full advantage of MLFFs, and (iii) establish a workflow that can be used in future applications of periodic CC calculations, where MLFFs are needed to bridge time- and system-size scales currently limited by the computational cost. The training data generated for the present study is made publicly available to serve as input data for other techniques.

## 2 Results

We employ the following notation throughout. ML­(*X*) refers to predictions made by an MLFF (QNEP or MACE) trained on data generated using method *X*. In the case of X = DFT_E_, the training data include energies computed using an approximate exchange and correlation density functional only. X = DFT_E,F_ also includes atomic forces in the training data. ΔML­(*X*) refers to results generated using a simple linear combination of two MLFFs. The first MLFF corresponds to ML­(DFT_E,F_), whereas the second MLFF is trained on the difference in energy between WFT method *X* and DFT. This includes Hartree–Fock (HF), Møller–Plesset perturbation theory (MP2), CC singles and doubles (CCSD) and CC singles, doubles, and perturbative triples (CCSD­(T)). More details are provided in section 4.

### 2.1 Diamond

We start by discussing the phonon dispersion of the carbon diamond. First, we focus on results generated using an MLFF trained on 200 training data points of DFT-PBE ground-state energies and forces. These data points have been randomly chosen from an MD trajectory, as described in section 4.3.3. Figure 1a) shows that ML­(DFT_E,F_) (green area) is able to satisfactorily reproduce the DFT phonon dispersions and harmonic approximation density of states (HA-DOS) (black dashed line).

**Figure 1:**
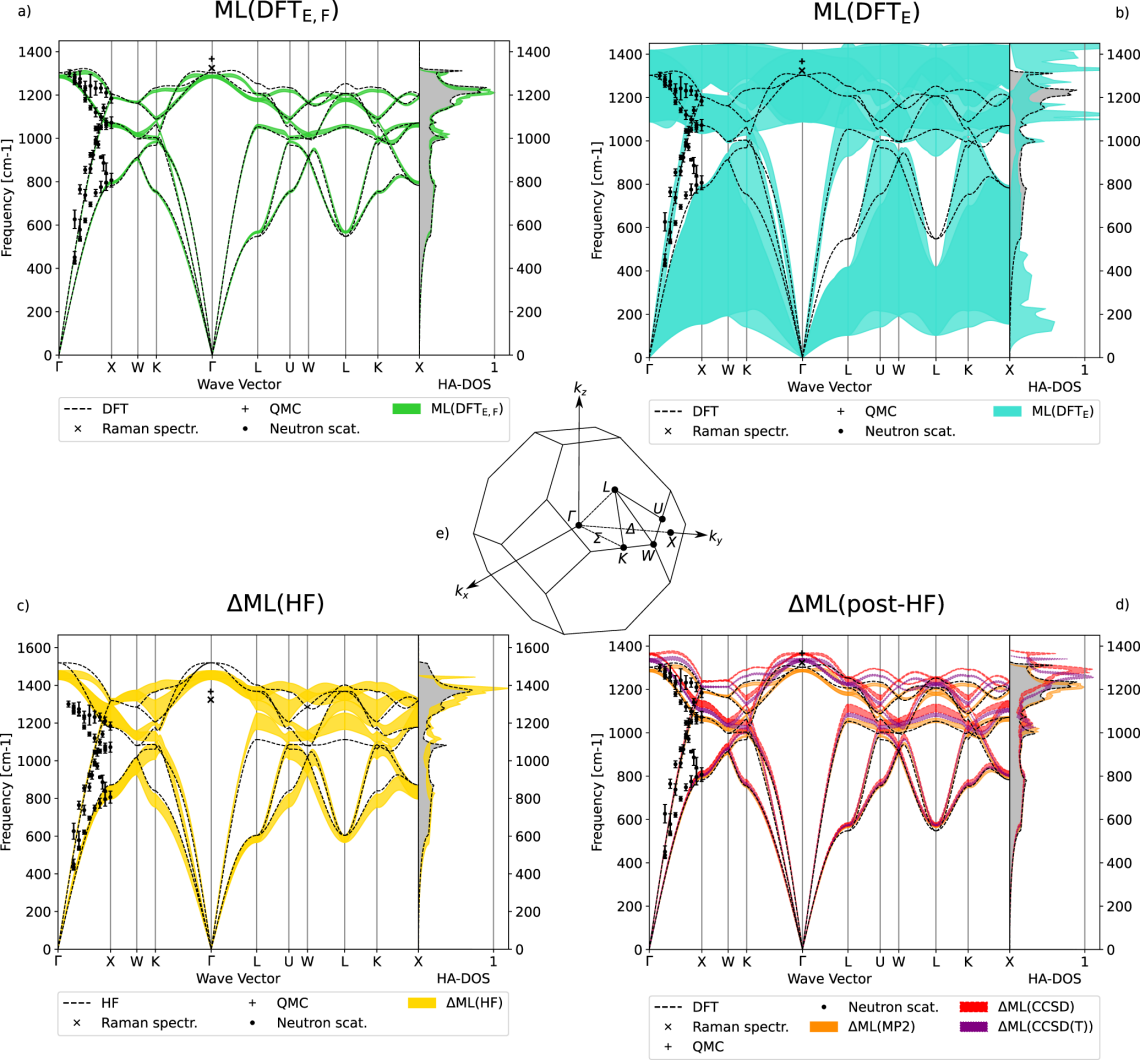
Phonon dispersions and DOS comparisons for diamond; neutron scattering data (black dots) from ref 32, Raman spectroscopy (black x) from ref 19, and quantum Monte Carlo data (black plus) from ref 18. (a) ML­(DFT_E,F_) (green area) vs DFT results (black dashed line). (b) ML­(DFT_E_) (trained only on E) (cyan area) vs DFT results (black dashed line). (c) ΔML­(HF) (yellow area) vs HF results (black dashed line). (d) Different ΔML­(WFT) results (gold, red dashed, purple dotted areas) vs DFT results (black dashed line). (e) Brillouin zone with relevant high symmetry points for diamond.

The green area depicts the maximum and minimum phonon frequency obtained for 5 different random seeds used in the training of the MLFFs. Note that the same randomly selected data points have been used during training. We refer to this disagreement as “seed based spread”. For a depiction of each individual seed, see section 1.3 of the SI. All seeds strongly overlap with the DFT results (black dashed line), except for at the L point, where ML­(DFT_E,F_) underestimates the upper optical and lower acoustic mode frequencies. This can be understood by noting that the MLFFs are trained on a 2 × 2 × 1 supercell of the conventional cell, where the L point is not represented (see Figure 1e; the L point points furthest “up”). Due to the relatively large computational cost of CCSD­(T) calculations, larger supercells could not be studied.

Next, we inspect an MLFF trained only on DFT energies and compared it to reference DFT results. In Figure 1b, it is clear that ML­(DFT_E_) (cyan area) cannot even qualitatively reproduce the phonon dispersions of DFT and exhibits notably larger seed-based spread, clearly demonstrating the need for atomic forces in the training data.

As a possible way to avoid the need for atomic forces for beyond-DFT methods, we explore a Δ-learning approach (described in detail in section 4.1). To verify that Δ-learning is able to reproduce phonon dispersions of the respective parent method, we look at ΔML­(HF), shown in Figure 1c (yellow area), as compared to a phonon dispersion at the level of HF theory (black dashed line). In contrast to ML­(DFT_E,F_) shown in Figure 1a, ΔML­(HF) results exhibit a stronger seed-based spread, illustrated by the broader yellow area. However, ΔML­(HF) still exhibits a relatively good qualitative agreement. The seed-based spread increases (thickening of the yellow area) at points where the model underestimates the HF reference data. This can be used to estimate the model’s reliability; the larger the seed-based spread, the less confident the prediction. In cases where ΔML­(HF) predictions deviate significantly from the base model ML­(DFT_E,F_), the seed-based spread increases; this is especially the case for optical modes. These discrepancies can be largely attributed to the potential energy surface of HF compared to DFT, particularly at the Γ-point, where it exhibits a stronger curvature. This is not well captured in the Δ-learning process, as it excludes forces at the HF level.

Having verified that our Δ-learning approach can be used to predict HF phonon dispersions, we now turn to post-HF methods. Figure 1d shows phonon dispersions at the level of ΔML­(MP2) (gold area), ΔML­(CCSD) (red dashed area), and ΔML­(CCSD­(T)) (purple dotted area). We first notice that the seed-based spread of these methods is smaller than for ΔML­(HF). This is attributed to the fact that these methods yield phonon frequencies closer to the ones obtained with DFT. We further corroborate this by observing that ΔML­(MP2), the result closest to DFT, exhibits lowest seed-based spread, in particular at the L point at around 1000 cm^–1^ (which also exhibits the largest seed-based spread overall).

The main differences between ΔML­(MP2), ΔML­(CCSD) and ΔML­(CCSD­(T)) are observed for the optical modes at high frequencies. ΔML­(CCSD) yields the highest frequencies for these modes and ΔML­(CCSD­(T)) is between DFT and ΔML­(CCSD). Compared to experimental findings obtained using neutron scattering^32^ (see Γ-X) and Raman spectroscopy^19^ (shown at Γ), ΔML­(CCSD­(T)) yields the most accurate results. This confirms expectations based on findings in molecular quantum chemistry,^8^ where CCSD­(T) also predicts vibrational frequencies with high accuracy. However, note that 200 single point CCSD­(T) calculations for the studied supercell consume about 750.000 cpuhs. For further comparison, Figure 1d depicts QMC data at Γ (black plus),^18^ which is also considered an accurate benchmark method and agrees with our ΔML­(CCSD) results. We note again that the highest acoustic modes at the L point features particularly large seed-based spread, indicating that these results are less reliable due to the limited supercell size. Section 1.3 of the SI shows all individual seed results explicitly.

We also investigated the dependence of the results discussed above on the size of the training data set. The corresponding phonon dispersions created by MLFFs trained only on around half the training data (98 points) can be found in section 1.1 of the SI; they are largely similar but show stronger seed-based spread (except for ML­(DFT_E_), where primarily the shape of the broadening changes to include imaginary modes).

### 2.2 Lithium Hydride

We now turn to the discussion of results obtained for the lithium hydride (LiH) solid. In this case, we use the local density approximation (LDA) for the exchange and correlation density functional, which was shown to agree better with experiment for lattice dynamic properties compared to DFT-PBE.^35^ The training data, including structures, energies and forces, for ML­(DFT_E,F_) were taken from an MD trajectory with 2000 steps. More details are provided in section 4.3.4. This number of configurations was necessary to achieve acceptable dispersions. Figure 2a shows that the phonon dispersion computed with ML­(DFT_E,F_) (green area) is mostly in good agreement with results generated by DFT (black dashed line). The acoustic modes show almost perfect agreement for all seeds, whereas the optical modes exhibit regions with significant discrepancies, especially when approaching the Γ point from K and L. These will be addressed in Figure 3. For a depiction of each individual seed, see section 2.3 of the SI.

**Figure 2:**
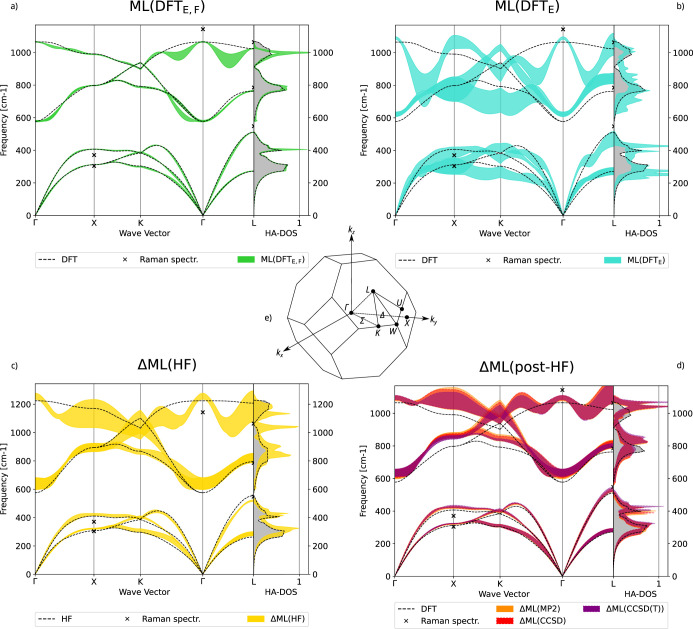
Phonon dispersions and DOS comparisons for LiH done with MACE; Raman spectroscopy data (black x) from ref 34. (a) ML­(DFT_E,F_) across various seeds (green area) vs DFT results (black dashed line). (b) ML­(DFT_E_) (trained only on E) across various seeds (cyan area) vs DFT results (black dashed line). (c) ΔML­(HF) across various seeds (yellow area) vs HF results (black dashed line). (d) Different ΔML­(WFT) results across various seeds (gold, red dashed, purple dotted areas) vs DFT results (black dashed line). (e) Brillouin Zone with relevant high symmetry points for cubic LiH.

**Figure 3:**
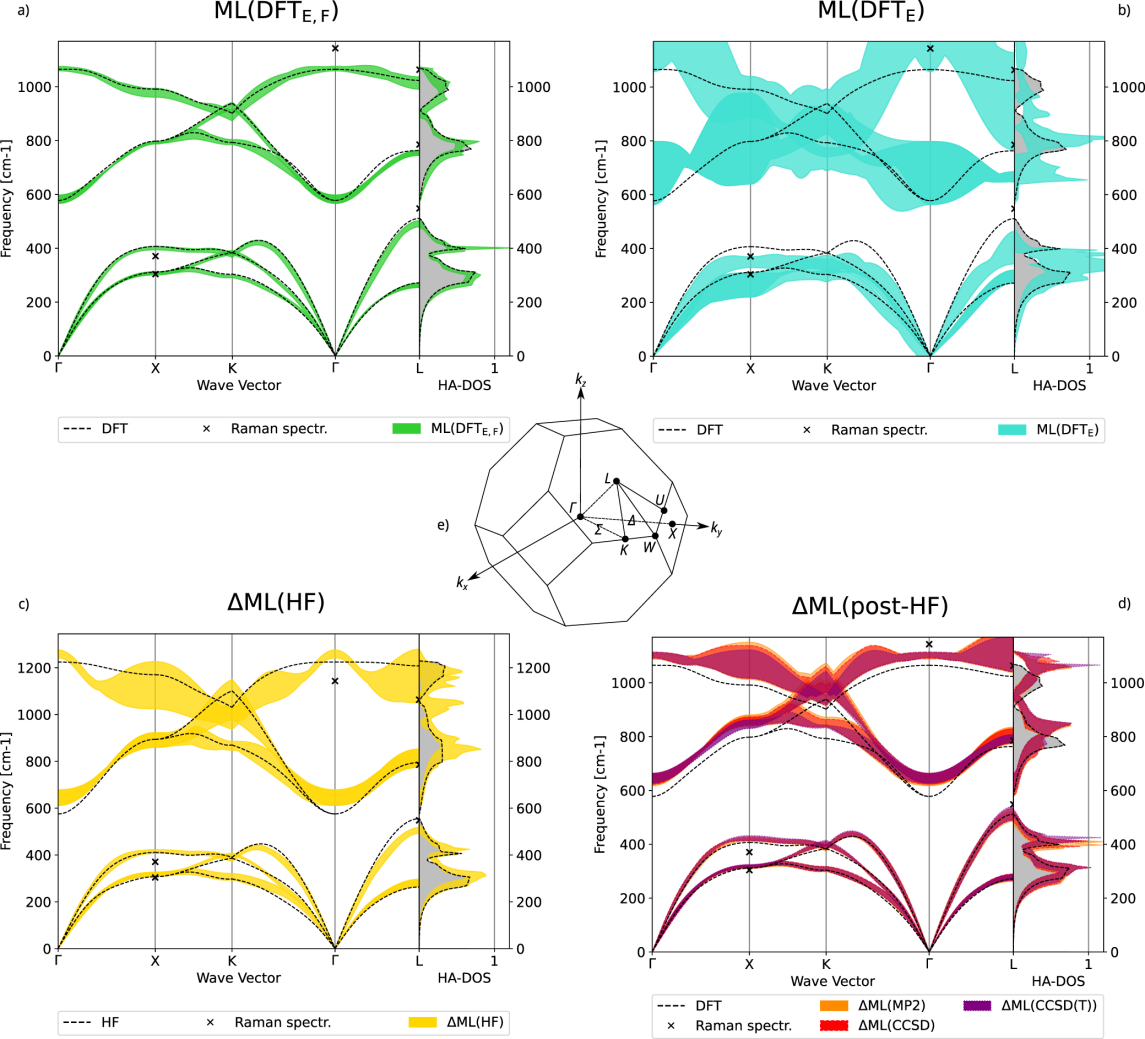
Phonon dispersions and DOS comparisons for LiH done with QNEP and ΔMACE; Raman spectroscopy data (black x) from ref 34. (a) ML­(DFT_E,F_) across various seeds (green area) vs DFT results (black dashed line). (b) ML­(DFT_E_) (trained only on E) across various seeds (cyan area) vs DFT results (black dashed line). (c) ΔML­(HF) across various seeds (yellow area) vs HF results (black dashed line). (d) Different ΔML­(WFT) results across various seeds (gold, red dashed, purple dotted areas) vs DFT results (black dashed line). (e) Brillouin Zone with relevant high symmetry points for cubic LiH.

Phonon frequencies at the Γ point are all in relatively good agreement, but it should be noted that this is partly a consequence of the fact that the splitting of the LO-TO (longitudinal and transversal optical) mode at this point is determined from a nonanalytic term correction (NAC) that is computed employing Born-effective charges at the level of DFT.^36^ This correction is identical for ML­(DFT_E,F_) and DFT phonon dispersions, explaining the perfect agreement of the splitting at Γ.

Figure 2b shows phonon dispersions generated by ML­(DFT_E_) (cyan area), which was trained using 200 configurations only as this is the number of systems that are feasible using periodic CCSD­(T) calculations without significant computational cost. Note that 200 single point CCSD­(T) calculations for the studied supercell consume about 10.000 cpuhs. Compared to DFT (black dashed line), ML­(DFT_E_) exhibits significant discrepancies especially for the optical modes. The only exception is again the Γ point due to inclusion of the NAC.

Following the same procedure as in the previous section, we then apply the Δ-learning approach to HF and compare with the reference HF phonon dispersion (black dashed line), depicted in Figure 2c (yellow area). The acoustic modes overlap quite well with just a slightly larger spread than ML­(DFT_E,F_). However, the agreement of the optical modes is much worse, although better than in the case of ML­(DFT_E_). Also notable is very large seed-based spread at the highest optical mode at the X point.

In Figure 2d, the post-HF Δ-learning approaches are plotted for MP2, CCSD and CCSD­(T) (gold, red dashed, purple dotted areas). Interestingly, all post-HF methods yield phonon dispersions that agree with each other relatively well for LiH. Note that the DFT-based NAC was also used in this case, which is slightly less justified for these methods. However, we stress that this correction only affects the splitting of the LO and TO modes at Γ. Since this splitting agrees relatively well between DFT and all post-HF methods at the other k-points, this approximation seems well justified, and finds some more legitimization in section 2.3. The capability to compute Born effective charges for the post-HF methods will be the topic of future work. Overall, we observe little seed-based spread in the acoustic modes, though slightly larger compared to ML­(DFT_E,F_). The seed-based spread again becomes very large for the optical modes. The largest spread can be seen in ΔML­(MP2), with ΔML­(CCSD­(T)) having the smallest. We observe again a particularly large seed-based spread at the X point. It is noteworthy that the frequencies of the optical modes are larger in all post-HF methods compared to DFT.

As before, the phonon dispersions generated by MLFFs trained on around half the training data (998 for ML­(DFT_E,F_), 85 for the ΔML­(WFT)) reveal, broadly speaking, similar performance, with stronger seed-based spread especially for Δ-learning results (see section 2.1 of the SI).

An attempt to mitigate the oscillatory behavior in the dispersion of the optical frequencies is found in Figure 3. Here, we used QNEP instead of MACE for the ML­(DFT_E,F_) and ML­(DFT_E_) results, the former also applied as the base for the Δ-learning results. The NN trained through QNEP includes long-range electrostatic interaction, reducing the oscillations, but increasing error and seed-based spread at the upper optical modes of the chosen high symmetry points.

None of the LiH phonon results - whether ML, DFT, or HF - reproduce the experimental Raman spectroscopy results^34^ particularly well. We will investigate how strong a role the anharmonicity of LiH^37^ plays here in section 2.3.

### 2.3 Velocity Autocorrelation Function

In addition to the phonon dispersions of diamond and LiH, Figures 1 and 2 show the vibrational densities of states in the harmonic approximation (HA-DOS). The MLFFs can also be used to estimate the density of states, including contributions beyond the harmonic approximation, by performing long MD simulations and computing the fast Fourier transform (FFT) of the velocity autocorrelation function (VACF) from the obtained trajectories. The vibrational density of states computed from the VACF is denoted as the VACF-DOS. To expedite this calculation process, only two of the five seeds from before are used to create the shown area spread for both HA-DOS and VACF-DOS.

We first apply this method to diamond. Figure 4 depicts the HA-DOSs as generated by DFT (black dashed line), ML­(DFT_E,F_) (green dashed area, Figure 4a), and ΔML­(CCSD­(T)) (purple dotted area, Figure 4b). As one could already observe in Figure 1d, these results are mostly similar, except for the optical modes, where ΔML­(CCSD­(T)) is shifted to higher frequencies compared to ML­(DFT_E,F_). To obtain the DOS including effects beyond the HA, we performed a molecular dynamics simulation (MD) at 300 K once using ML­(DFT_E,F_) (green area, Figure 4a) and once using ΔML­(CCSD­(T)) (brown area, Figure 4b). The VACF-DOSs were obtained by a FFT of the VACFs. However, the peak positions in both VACF-DOSs are similar to those in the respective HA-DOS. The remaining discrepancies in peak height and shape are due to the finite mesh and supercell sizes used in the VACF calculations. Although MLFFs can be used to generate MD trajectories efficiently, it is still impossible to perform calculations of VACF-DOSs using supercells that correspond to the dense meshes used for the HA-DOSs.

**Figure 4:**
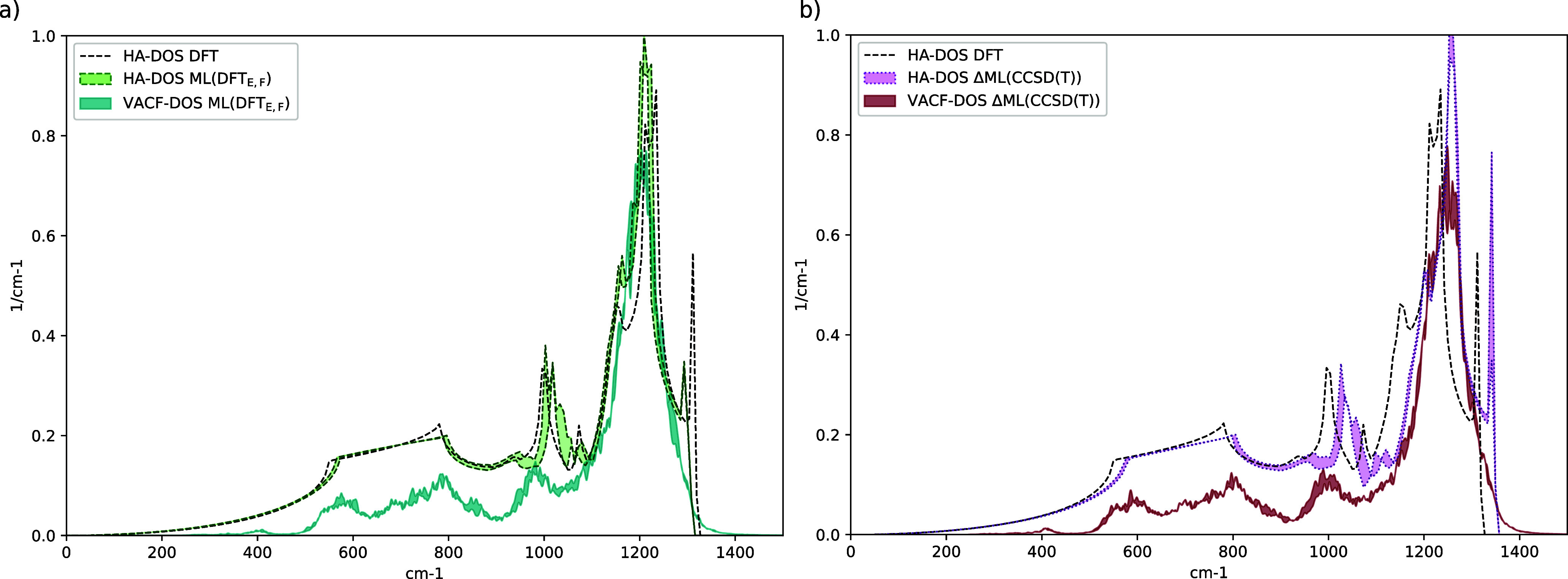
HA- and VACF-DOS comparisons for diamond. (a) Comparison between DFT HA-DOS (black dashed line), ML­(DFT_E,F_) HA-DOS (green dashed area), and ML­(DFT_E,F_) VACF-DOS (teal area). (b) Comparison between DFT VDOS (black dashed line), ΔML­(CCSD­(T)) HA-DOS (purple dotted area), and ΔML­(CCSD­(T)) VACF-DOS (brown area). HA-DOS was calculated as explained in section 4.3, using either ML or DFT methods; areas were created using two of the five seeds from section 2.1. VACF was generated using ML MDs at 300 K.

Next, we turn to LiH to see whether anharmonic effects have a larger contribution to the vibrational properties. In Figure 5, the same procedure as before is plotted for LiH. The HA-DOS generated by DFT (black dashed line), ML­(DFT_E,F_) (green dashed area, Figure 5a) and ΔML­(CCSD­(T)) (purple dotted area, Figure 5b) largely overlap, with ΔML­(CCSD­(T)) exhibiting a shift to higher frequencies in the optical modes (as seen before in Figure 2d). The VACF-DOSs are once again calculated from an MD run using ML­(DFT_E,F_) (green area, Figure 5a) and ΔML­(CCSD­(T)) (purple area, Figure 5b), this time at 20 K to match with the neutron scattering experiment shown in Figure 6. Note that this temperature should be sufficient to excite all vibrations in the studied frequency range. However, the VACF-DOSs and HA-DOSs once again largely overlap, showing that direct anharmonic effects do not have a strong effect on the DOS. Looking at the peak originating from the optical modes at high frequencies in Figure 5b, this also legitimizes the use of the DFT Born charges for the ΔML­(CCSD­(T)) calculations: The VACF-DOS is Born-charge independent, yet the two overlap at this peak (the only one affected by the nonanalytical term correction).

**Figure 5:**
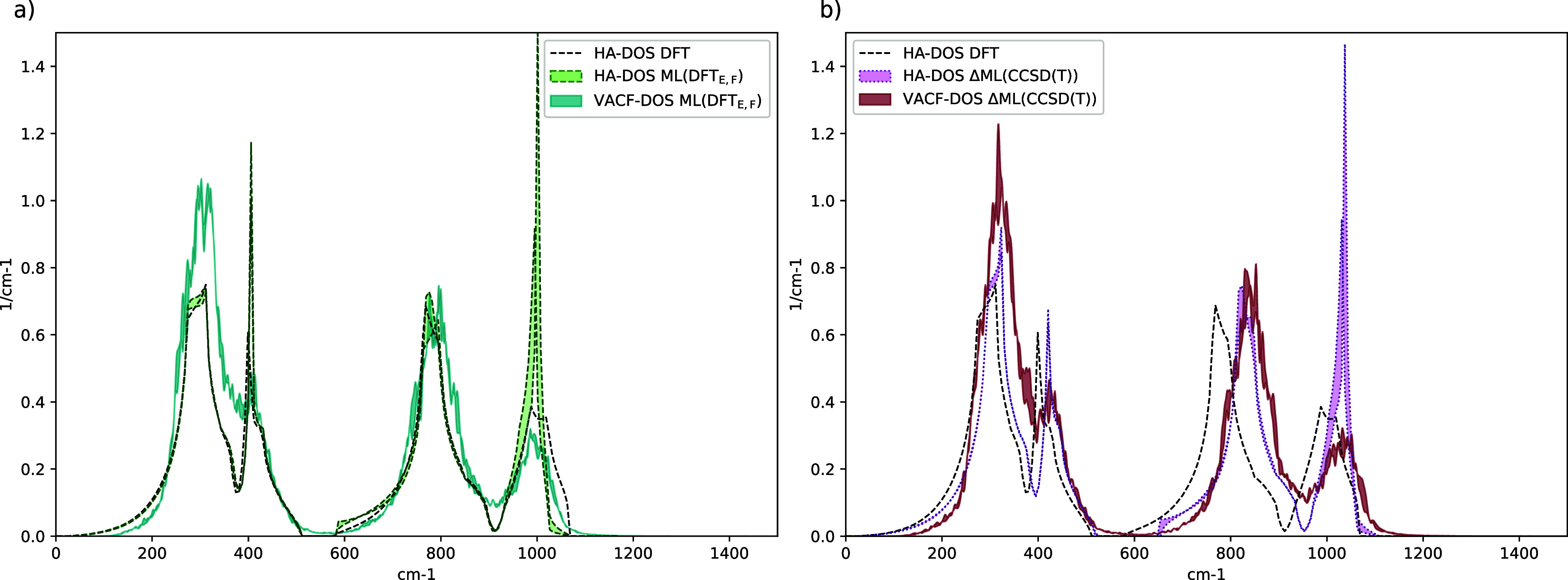
HA- and VACF-DOS comparisons for LiH. (a) Comparison between DFT HA-DOS (black dashed line), ML­(DFT_E,F_) HA-DOS (green dashed area), and ML­(DFT_E,F_) VACF-DOS (teal area). (b) Comparison between DFT HA-DOS (black dashed line), ΔML­(CCSD­(T)) HA-DOS (purple dotted area), and ΔML­(CCSD­(T)) VACF-DOS (brown area). HA-DOS was calculated as explained in section 4.3, using either ML or DFT methods; areas were created using two of the five seeds from section 2.2. VACF was generated using ML MDs at 20 K.

**Figure 6:**
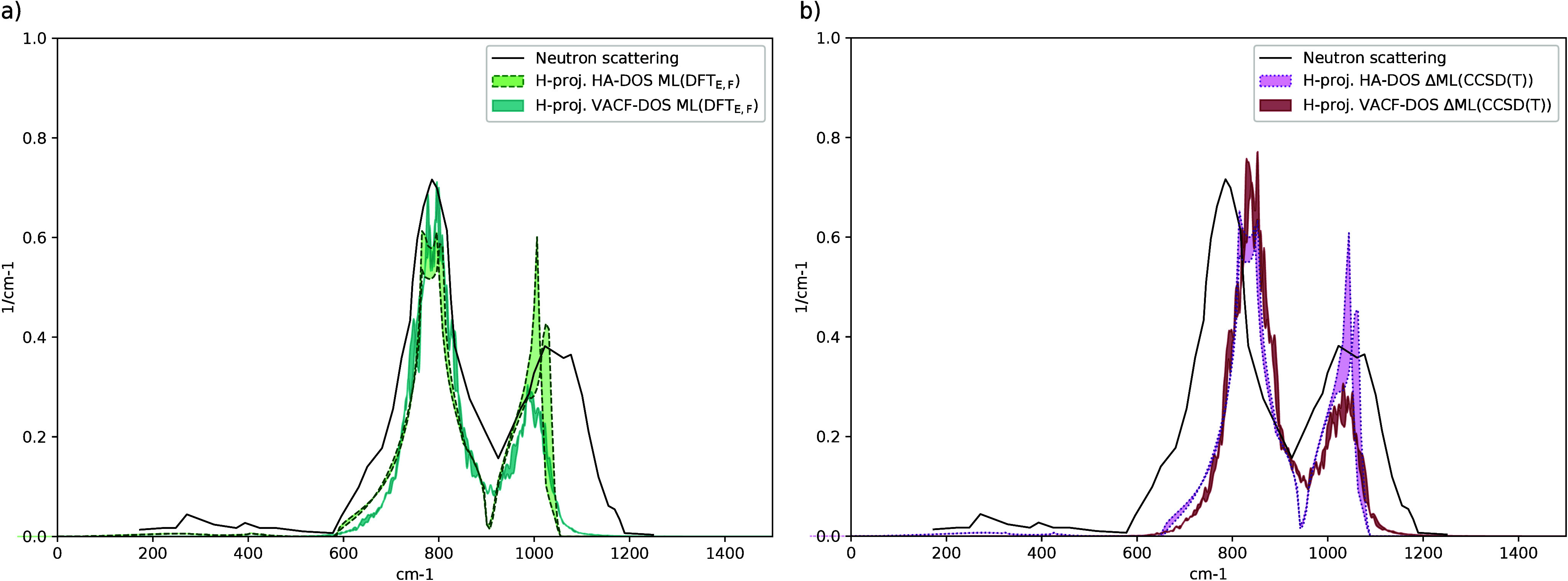
H-projected HA- and VACF-DOS comparisons for LiH. (a) Comparison between H-projected neutron scattering DOS^38^ (black line, scaled up for better visibility), H-projected ML­(DFT_E,F_) HA-DOS (green dashed area), and H-ptojected ML­(DFT_E,F_) VACF-DOS (teal area). (b) Comparison between H-projected neutron scattering DOS^38^ (black line, scaled up for better visibility), H-projected ΔML­(CCSD­(T)) HA-DOS (purple dotted area), and H-projected ΔML­(CCSD­(T)) VACF-DOS (brown area). HA-DOS was calculated as explained in section 4.3, using ML methods. Spreads are given by two different seed results. VACF was generated using ML MDs at 20 K.

In Figure 6, we show a comparison between experimental neutron scattering data from ref 38 (black line, scaled up for better visibility) and the H-projected (HA/VACF-)­DOSs from Figure 5. We find that the experimental peak around 800 cm^–1^ matches well with the ML­(DFT_E,F_) results, the experimental peak around 1100 cm^–1^ is in better agreement with the corresponding peaks from ΔML­(CCSD­(T)).

Remaining errors may stem from the experiments, mismatches in lattice constants between experiments and training data, and finite-size effects. Additionally, nuclear quantum effects may also be significant.

## 3 Discussion

In this work, we have used Δ-learning to obtain MLFFs for periodic solids at MP2, CCSD, and CCSD­(T) levels of accuracy. An advantage of the employed Δ-learning approach is that it circumvents the need for calculating atomic forces at the desired level of quantum chemical many-electron theories, such as MP2, CCSD or CCSD­(T) theory. Instead, the presented approach combines a more precise MLFF trained on DFT energies and forces, with an MLFF trained on MP2, CCSD or CCSD­(T) energies only. We have assessed the precision of the (Δ-learning) MLFFs by comparing to (HF) DFT reference results using different seeds for MACE. Throughout this procedure, identical training data structures were used. The accuracy of the MLFFs at the level of DFT and CCSD­(T) theory was assessed by comparing to experimental vibrational frequencies. We have also demonstrated that training MLFFs on total energies alone, as would be desired for methods where atomic forces are not already implemented, does not yield very precise force fields. Reducing the training data set size does not lead to significant deterioration of results, as shown in sections 1.1 and 2.1 of the SI.

In the case of carbon diamond, we found that the acoustic phonon dispersions agree very well with experimental findings at both the DFT-PBE and CCSD­(T) levels of theory. ML­(DFT_E,F_) is capable of reproducing the DFT results with high precision. The seed-based spread of ΔML is sufficiently small to differentiate the computed phonon frequencies between DFT-PBE, MP2, CCSD, and CCSD­(T). For the optical modes, we find that CCSD­(T) theory yields higher frequencies in better agreement with experiment compared to DFT-PBE.

Our findings for LiH are partly similar to those for diamond. For the optical modes, the CCSD­(T) theory yields higher frequencies than DFT-PBE. Moreover, the good agreement between MP2, CCSD, and CCSD­(T) theories indicates that the employed level of electronic structure theory yields highly reliable potential energy surfaces. In contrast to the results for diamond, we find for LiH that long-range electrostatic effects play an important role in the training of precise MLFFs, regardless of the level of theory. In particular, we found that disregarding long-range effects leads to unphysical oscillations in the phonon frequency dispersion of optical modes. We have mitigated this behavior using QNEP at the level of ML­(DFT_E,F_).

Using ML­(DFT_E,F_) and ΔML­(CCSD­(T)), it is possible to perform MD simulations for relatively long time scales needed to compute converged VACFs (30000 steps for cells containing 512 atoms, see Figure 7 and Table 1). Our calculations show that anharmonic effects yield only small changes in the VDOS. However, both DFT-LDA and CCSD­(T) phonon dispersions as well as vibrational densities of states in the harmonic approximation do not match the experimental findings well. Investigating H-projected vibrational densities that can be directly compared to neutron scattering yields better results at the DFT-LDA level for lower optical modes, while for the higher optical modes the CCSD­(T) level fares best. The remaining discrepancies between theory and experiment cannot be fully explained and may necessitate inclusion of effects from lattice expansion and perhaps nonadiabaticity. Additionally, nuclear quantum effects could also play a significant role. This requires further investigation beyond the scope of the present work. However, The published training data and the outlined ΔML approach can form the basis of future work employing path integral molecular dynamics

**Figure 7:**
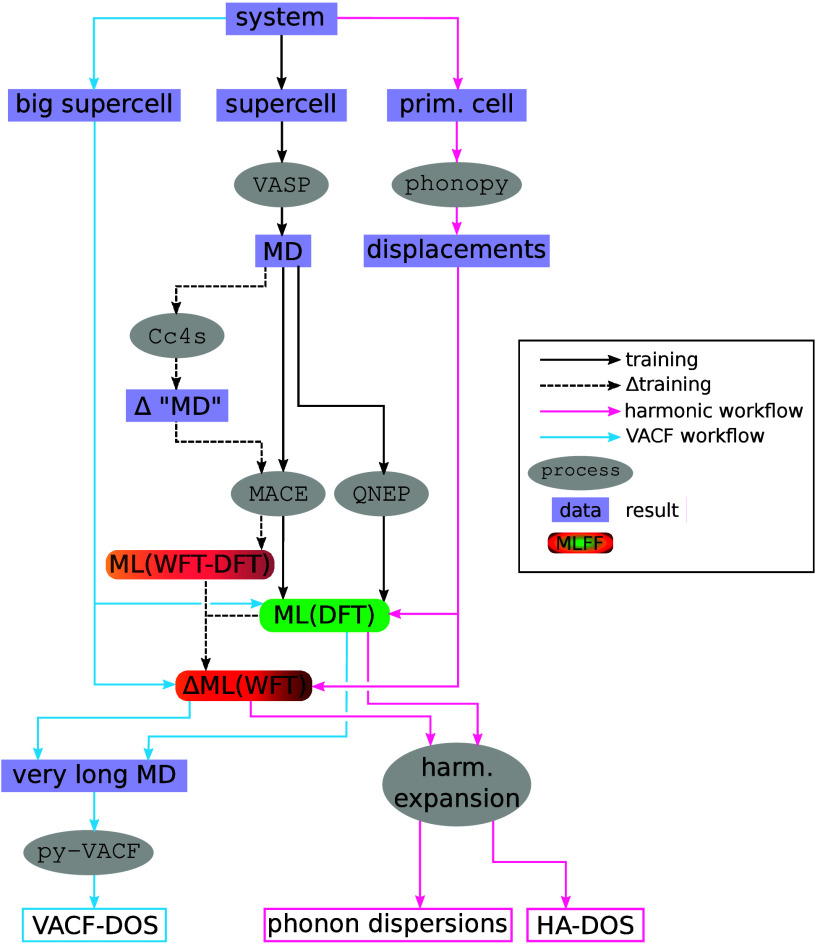
Workflow for this paper. First, a system (diamond or LiH) is chosen. In the black branch, a supercell of the conventional cell is created (for exact settings always see Table 1) using VASPKIT.^39^ From this initial configuration, VASP^40–42^ is used to create an MD. MACE^27,28^ or QNEP^29–31^ is trained on said MD, yielding ML­(DFT). In the black dashed branch, 200 steps of the previous MD are then fed into Cc4s,^43^ which generates WFT level energies for each configuration (and forces in the case of HF). The energy *differences* from this Δ“MD” is again used to train MACE, yielding ML­(WFT-DFT); adding predictions from ML­(WFT-DFT) to ML­(DFT_E,F_) creates the results from ΔML­(WFT). In the pink branch, a relaxed primitive cell is fed into phonopy,^44,45^ yielding displacement configurations. For these, the MLFFs predict energies and forces, which are then interpreted by phonopy for a harmonic expansion to generate the phonon dispersions and HA-DOS. In the light blue VACF branch, the ML­(DFT_E,F_) and ΔML­(CCSD­(T)) are both used to create two very long MDs, which are handed to py-VACF^46^ to yield the VACF-DOS.

**Table 1:** Settings for Different Parts of the Workflow^a^

	C	LiH
supercell	2 × 2 × 1 (32)	2 × 2 × 2 (64)
*n* [MD, ML(DFT_E,F_)]	200	2000
*n* [Δ“MD”, ML(WFT-DFT), ML(DFT_E_)]	200	200
r_max (MACE)	3.0 Å	9.0 Å
cutoff (QNEP)		8.0, 4.0 Å
big supercell	4 × 4 × 4 (512)	4 × 4 × 4 (512)
*n* [very long MD]	30000	30000
*T* [very long MD]	300 K	20 K
supercell [displacements]	4 × 4 × 4 (128)	4 × 4 × 4 (128)
mesh [harm. expansion]	24 × 24 × 24	24 × 24 × 24

a Refer back to Figure 7. *n* is the number of MD steps, r_max defines up to what distance atoms are included in the descriptor, and *T* is temperature.

In all simulations, the computational bottleneck remains the generation of training data at higher levels of theory. In particular, the sizes of the simulation supercells are the main limitation. In the case of asymmetric supercells, e.g., carbon diamond, we find that this leads to lower precision of the respective MLFFs at certain *k*-points.

Taken together, our findings indicate that improving MLFFs by incorporating forces also at a higher level of theory is particularly beneficial compared to relying solely on the availability of single-point energies, even when the latter can be computed using larger simulation cells. We therefore conclude that implementations including forces are highly likely to offer substantial advantages despite the associated increase in computational cost. Consequently, we are pursuing the implementation of forces within periodic CCSD­(T), which is currently underway. In addition, our results suggest that investigating architectures that explicitly account for long-range interactions represents a particularly promising direction for future work.

## 4 Methods

This section summarizes details of all employed methods and is organized as follows. Section 4.2 explains the general workflow used to obtain the vibrational density of states and phonon dispersions for the materials studied in this work. Section 4.1 describes the Δ-learning approach needed to compute vibrational properties at the level of HF, MP2, CCSD and CCSD­(T) theory. Section 4.3 summarizes details of the electronic structure theory calculations used to generate the training data as well as settings used to train the MLFFs.

### 4.1 Δ-Learning

We first explain additional details of the Δ-learning approach used to obtain MLFFs at the level of wave function theories. Here, wave function theory (WFT) stands for Hartree–Fock (HF), second-order Møller–Plesset perturbation (MP2), coupled-cluster singles and doubles (CCSD) or coupled-cluster singles, doubles plus perturbative triples (CCSD­(T)) theory. The Δ-learning approach is necessary because the implementation of periodic MP2 and CC theory used in the present work does not yet provide atomic forces. MLFFs trained on total energies alone do not achieve the level of precision needed for reliable phonon dispersions and VDOSs, as shown in Figures 1–3b. To overcome this limitation, the Δ-learning approach combines training on DFT energies and forces with training on differences between energies obtained at the levels of DFT and WFT. We use a Δ-learning approach, which involves training two MLFFs:

1. ML­(DFT_E,F_) refers to an MLFF trained on DFT energies (E) and atomic forces (F).
2. ML­(WFT-DFT) refers to an MLFF trained on the difference in E between structures calculated by DFT and the relevant WFT-based method, Δ*E* = *E*_WFT_ – *E*_DFT_.

We employ MACE or QNEP to train ML­(DFT_E,F_) by providing xyz files containing the structural information for a set of supercells with corresponding total energies and atomic forces. These structures are taken from an MD trajectory. System-specific details of the MD simulations are summarized in Table 1. More details about the settings used by MACE and QNEP during training are specified in Section 4.3.1.

ML­(WFT-DFT) is trained using MACE by providing corresponding xyz-files that contain the structural information along with the corresponding total energy differences, Δ*E* = *E*_WFT_ – *E*_DFT_.

To predict energies at the level of WFTs for a given structure, we simply add the predicted energies of ML­(DFT_E,F_) and ML­(WFT-DFT):

1 $$E_{\Delta ML\left(WFT\right)}=E_{ML\left(DFT_{E,F}\right)}+E_{ML\left(WFT-DFT\right)}$$

The atomic forces at the level of WFTs are also obtained by adding the predicted atomic forces of both MLFFs. Note that, as a shorthand, we always refer to the total result as ΔML­(WFT) (see the black dashed arrow branch in Figure 7): It is the result of adding ML­(WFT-DFT) (sheer yellow-red gradient rounded rectangle) to ML­(DFT_E,F_) (green rounded rectangle).

In this work, we train ΔML­(WFT) models for HF, MP2, CCSD, and CCSD­(T). ΔML­(HF) also serves as a “sanity check”, as we can produce HF-level phonon dispersions using VASP without ML, and compare to ΔML­(HF) (see Figure 1–3c). The other Δ-learning results are found in Figure 1–3d.

### 4.2 Workflow

A workflow of the data and software used is depicted in Figure 7. In addition, a summary of the most relevant settings is given in Table 1, containing information about the employed cell sizes and the computational parameters.

The black arrows in Figure 7 depict the flow of data for training MLFF. After choosing a system (diamond or LiH), a selected supercell is generated using, in our case, VASPKIT^39^ (for details see Table 1) and used to perform MD simulations at the level of DFT-PBE (diamond) or DFT-LDA (LiH) using VASP.^40–42^ The MD trajectory including computed energies and forces is fed into MACE^27,28^ (or, in the case of Figure 3, QNEP^29–31^ ) for training and, still following the black arrows, produces ML­(DFT). ML­(DFT_E,F_) and ML­(DFT_E_) refers to training with MACE or QNEP on both energies and forces or energies alone, respectively.

Black dashed arrows in Figure 7 illustrate the Δ-learning approach used in this work. We employ a subset of structures from the DFT MD to compute MP2, CCSD and CCSD­(T) energies. The CC calculations are performed using Cc4s.^43^ The previously generated DFT energies for the same structures are subtracted to yield Δ‘MD” training data, which is used in MACE to train ML­(WFT-DFT). WFT stands for HF, MP2, CCSD or CCSD­(T). ΔML­(WFT) is a MLFF, denoting predictions from ML­(WFT-DFT) and ML­(DFT_E,F_) added together. For further details on the Δ-learning process see section 4.1.

The workflow used to compute phonon dispersions and VDOSs in the harmonic approximation is outlined with pink arrows. First, a relaxed primitive cell of the relevant system is fed into phonopy,^44,45^ which yields structures for different displacements. For these, the MLFFs predict energies and forces, which are fed back into phonopy to compute the phonon dispersions and HA-DOS shown in Figures 1 (diamond) and 2 (LiH) (pink boxes in Figure 7). In the case of LiH, we also include the NAC (not shown in Figure 7).

In the VACF-DOS workflow (blue arrows), a big supercell (exact settings can be found in Table 1) is used as the initial configuration for a very long MD generated by using the MLFFs. The atomic velocities of the very long MD are used to compute autocorrelation functions, which are Fourier-transformed by py-VACF,^46^ yielding the VACF-DOS found in Figures 4, 5, and 6 (blue box in Figure 7).

### 4.3 Computational Details

#### 4.3.1 MACE

The majority of MLFFs presented in this work are based on the implementation of the message-passing neural network MACE.^27,28^ In the training procedure, five randomly chosen seeds (5192, 5391, 5735, 7271, and 861) were employed. The optimization process took 50 epochs. The MLFFs were used to compute phonon dispersions and HA-DOSs. In the case of the more expensive VACF MD needed for VACF-DOSs, only 5735 and 861 were used. For a depiction of each phonon dispersion for each model and seed, see sections 1.3 (diamond) and 2.3 (LiH) of the SI . Table 1 summarizes the computational parameters; for example, the supercell size and the radial cutoff parameter (r_max) within which atoms are considered for a single descriptor. Note that the values for hyperparameter r_max were 3 and 9 Å for diamond and LiH, respectively. The larger cutoff for LiH is necessary because its ionic bond character substantially increases long-range interatomic interactions compared to diamond.

To investigate the dependence on the training data set size we also trained MLFFs with half the training data set size. “Half” sets are defined such that they contain only 98 randomly picked structures for training, whereas the remaining 102 are used for testing. These results are found in Sec 1.1 (2.1 for LiH) of the SI. For the RMSEs, see also section 1.2 (2.2 for LiH) of the SI. Overall, half sets showed around equal agreement compared to full sets with partially increased seed-based spread. The RMSEs for all sets and methods in are in the meV/atom range, except ML­(DFT_E_) for diamond, which is in the 10 meV/atom range.

#### 4.3.2 QNEP

To investigate the role of long-range forces, MLFFs based on QNEP^29–31^ were also employed, specifically in Figure 3. Here, too, five random seeds were used, although they cannot be specifically chosen in the QNEP architecture, but are randomly generated every time a new training run is started. Default settings were used, most importantly 8.0 and 4.0 Å radial and angular cutoffs, respectively, and charge_mode 2, referring to using partial charges from the reciprocal space only.

#### 4.3.3 Diamond

The training data structures for diamond are obtained as follows: We use VASP^40–42^ to perform an MD simulation at the level of DFT-PBE for a 32 atom starting structure (2 × 2 × 1 supercell of the conventional basis cell, lattice constant 3.561 Å) to produce a 2000 step MD trajectory. In order to ensure structural variety in our training structures, we applied a temperature sweep from 0 to 500 K employing a Langevin thermostat.

A randomly chosen subset of 200 of these training structures are used to perform single-point HF and post-HF calculations. The HF calculations of the supercell employ a 3 × 3 × 3 k-point mesh and a plane wave basis energy cutoff of 400 eV. The CCSD and CCSD­(T) calculations are performed using the Cc4s code^43^ interfaced to VASP. In the CC calculations, 10 frozen unoccupied natural orbitals per occupied state were used. Furthermore, the post-HF calculations only sample the Γ-point of the first Brillouin zone of the supercell. To correct for the basis-set-incompleteness and finite-size errors of the correlation energies, we employ approximate corrections summarized in refs 47 and 48 also used in recent related work.^13^ The WFT training data is generated by selecting 200 structures randomly from the original VASP MD, and computing total energies for each one. The DFT energies for said structures are then subtracted, so only the difference of the energies will be used in training.

In the case of diamond, ML­(DFT_E,F_) was trained on the same 200 structures as ΔML­(WFT) (instead of the full 2000) as this turned out to be sufficient.

For the calculations of harmonic phonons and related properties, we use phonopy^44,45^ with a 4 × 4 × 4 supercell (of the primitive basis cell, so 128 atoms); for the HA-DOS comparison, we use a 24 × 24 × 24 mesh. To generate primitive basis cells for our structures, we use VASPKIT.^39^

For the calculation of the VACFs, we used the MACE calculator for ASE^49,50^ to create an MD of 30000 steps at 300 K, based on a 512 atom (4 × 4 × 4) starting structure; we did this once using ML­(DFT_E,F_), and once using ΔML­(CCSD­(T)). The resulting MD was fed into the VACF code taken from ref 46.

#### 4.3.4 Lithium Hydride

The procedure for LiH largely followed that for C in section 4.3.3, with the same codes used. A lattice constant of 4.017 Å was used for LiH. Unlike diamond, interatomic interactions in LiH are dominated by ionic bonds, causing long-range effects. Thus, a bigger supercell (2 × 2 × 2, 64 atoms) was necessary in the training data generation, and for ML­(DFT_E,F_) *all* 2000 MD steps were used in training. We note that using half the number of training points does not change results, as shown in section 2.1 of the SI. The VASP calculations were done using DFT-LDA. As ML­(DFT_E_) represents our WFT test case, it was trained on 200 randomly chosen structures.

## Supplementary Material



## Data Availability

All data generated or analyzed during this study are included in this published article and its Supporting Information files. The data sets used and/or analyzed during the current study are available at ref 51. The Cc4s documentation and installation guide can be found at https://manuals.cc4s.org/user-manual/. The MACE documentation and installation guide can be found at https://mace-docs.readthedocs.io/en/latest/. The QNEP documentation and installation guide can be found at https://gpumd.org/nep/index.html.

## Notes

RMSEs, phonon dispersions trained on half the data, and individual seed lines for full set phonon dispersions for both systems (diamond, LiH) and methods (MACE alone, MACE and QNEP) (PDF)
