# Supplementary material for: Machine-Learned Force Fields for Lattice Dynamics at Coupled-Cluster Level Accuracy
Source: J Chem Theory Comput. 2026 Jul 1;22(13):6906–16. doi: 10.1021/acs.jctc.6c00854 (PMC13374045; doi:10.1021/acs.jctc.6c00854)
Supplement: Supplementary file 1 [file ct6c00854_si_001.pdf]

Machine-Learned Force Fields for Lattice Dynamics at Coupled-Cluster Level Accuracy

Sita Schönbauer[1*], Johanna P. Carbone[2,1], Fredrik V. Eriksson[1], Florian Libisch[1], Andreas Grüneis[1]

[1*]Institute of Theoretical Physics, Technical University of Vienna, Wiedner Hauptstraße 8–10, 1040 Vienna, Austria.

[2]Faculty of Physics and Center for Computational Materials Science, University of Vienna, Kolingasse 14-16, 1090 Vienna, Austria.

*Corresponding author(s). E-mail(s): sita.schoenbauer@tuwien.ac.at;

Keywords: Machine Learning, Phonons, Coupled Cluster, Density Functional Theory

1 Diamond

1.1 Half Sets

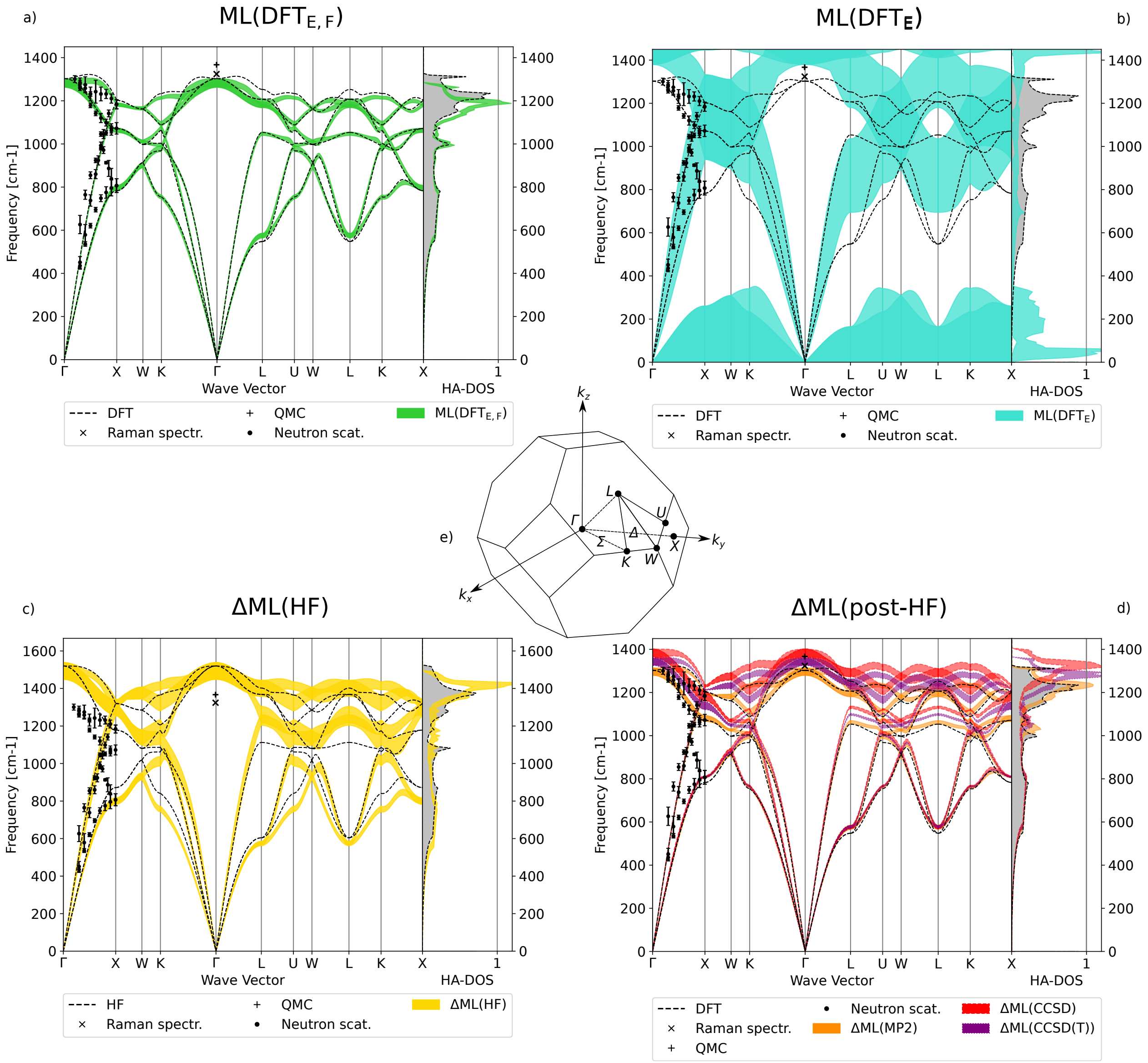

Fig. S.1 Phonon dispersions and DOS comparisons for diamond using only 98 data points for training; Neutron scattering data (black dots) from [1], Raman spectroscopy (black x) from [2] and Quantum Monte Carlo data (turquoise plus) from [3]. a) ML(DFT$_{E,F}$) (green area) vs. DFT results (black dashed line). b) ML(DFT$_E$) (trained only on E) (cyan area) vs. DFT results (black dashed line). c) ΔML(HF) (yellow area) vs. HF results (black dashed line). d) Different ΔML(WFT) results (gold, red dashed, purple dotted areas) vs. DFT results (black dashed line). e) Brillouin Zone with relevant high symmetry points for diamond.

1.2 RMSEs

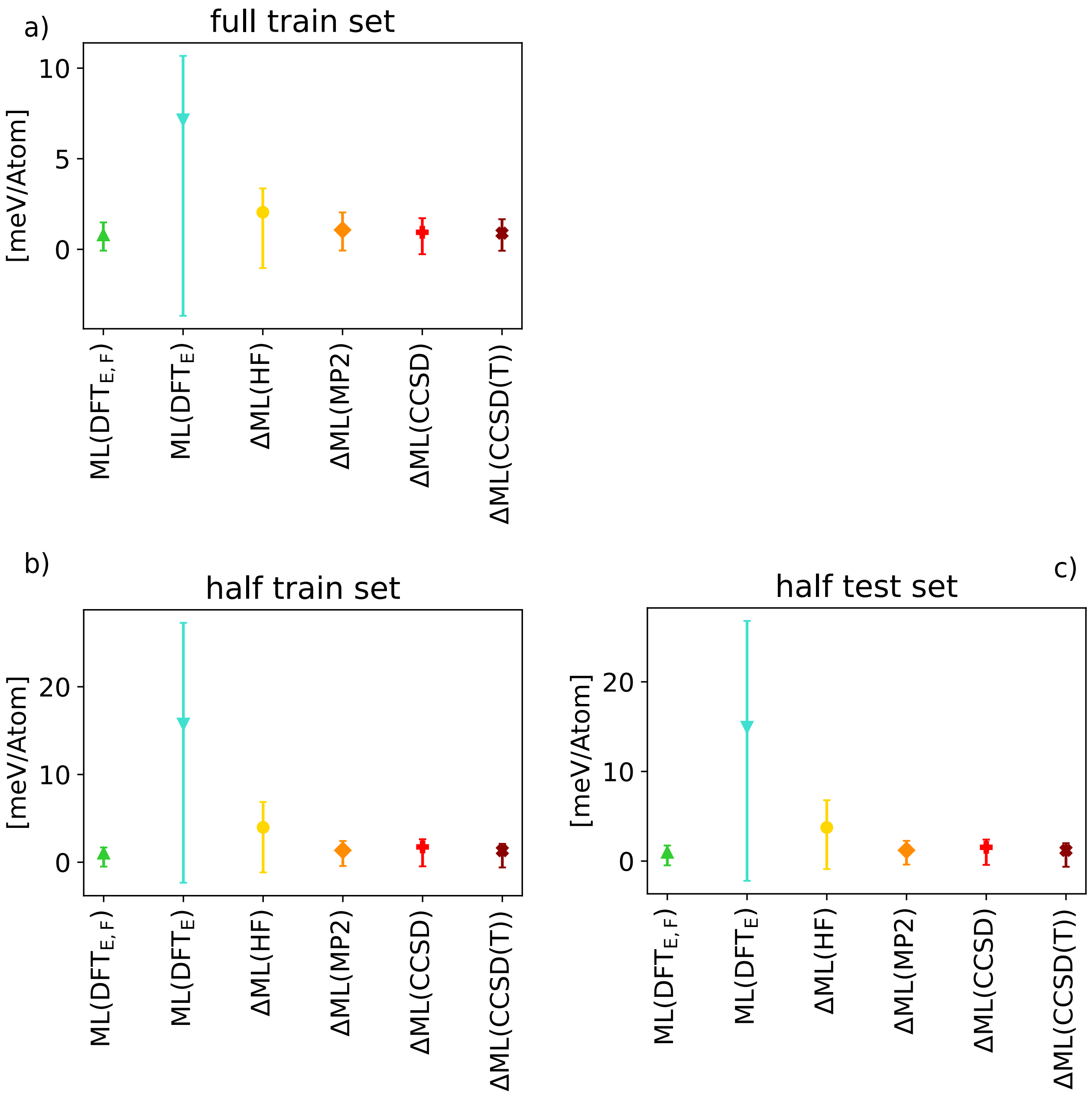

Fig. S.2 RMSEs for full train set (a), half train set (b) and half test set (c) for various MLFFs. ΔMLs always refer to errors from the combination of ML(DFT$_{E,F}$) and ΔML. Spreads in error represent the different seeds.

1.3 Seed lines

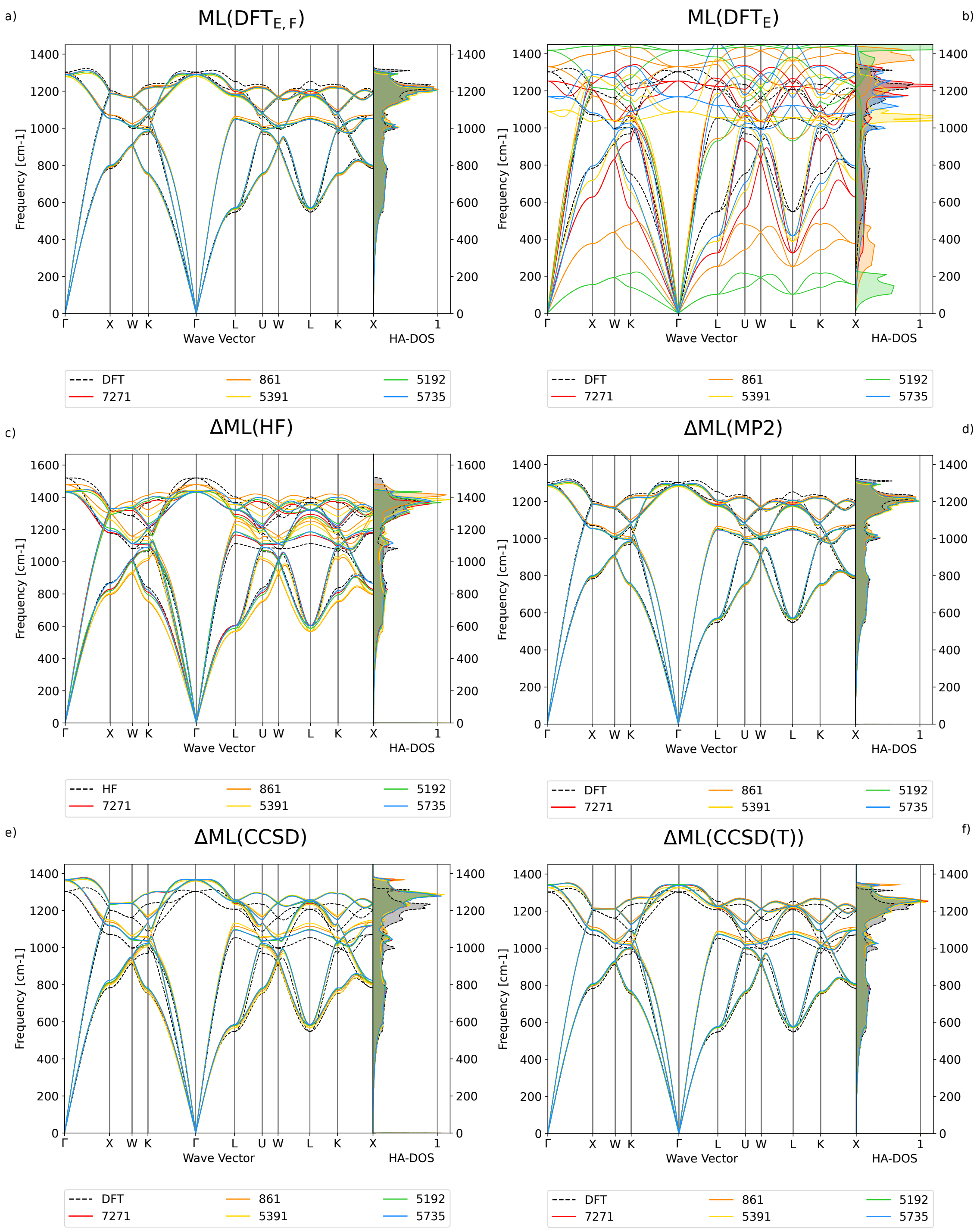

Fig. S.3 Diamond Phonons showing individual lines for each seed (red, orange, yellow, green, blue lines) for ab initio DFT (black dashed line) and a) ML($DFT_{E,F}$) b) ML(DFT_E) c) ΔML(HF) d) ΔML(MP2) e) ΔML(CCSD) f) ΔML(CCSD(T)).

2 Lithium Hydride

2.1 Half Sets

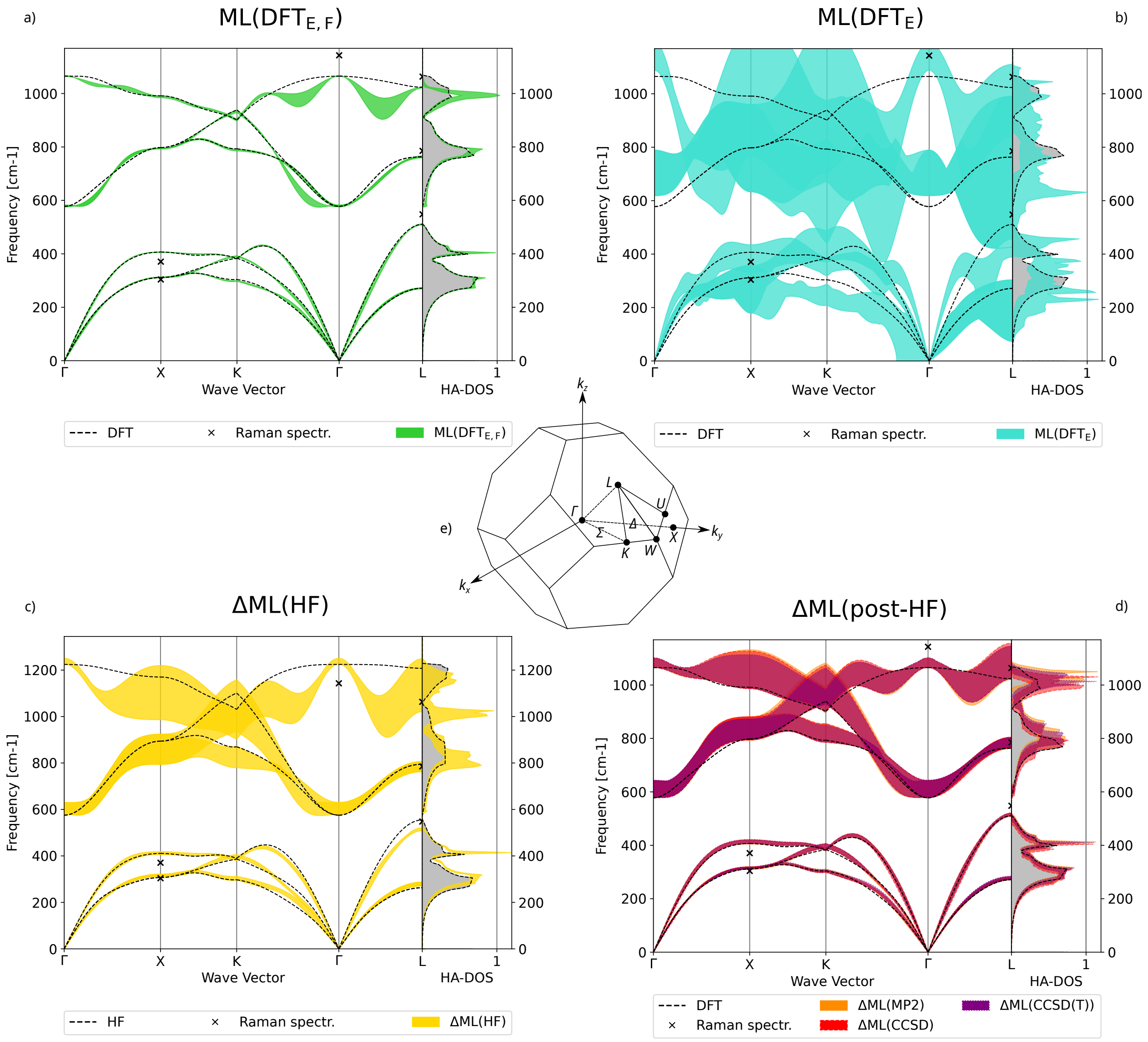

Fig. S.4 Phonon dispersions and DOS comparisons for LiH using 998 training points for ML(DFT$_{E,F}$) and 98 for the rest; Raman spectroscopy data (black x) from [4]. a) ML(DFT$_{E,F}$) across various seeds (green area) vs. DFT results (black dashed line). b) ML(DFT$_E$) (trained only on E) across various seeds (cyan area) vs. DFT results (black dashed line). c) ΔML(HF) across various seeds (yellow area) vs. HF results (black dashed line). d) Different ΔML(WFT) results across various seeds (gold, red dashed, purple dotted areas) vs. DFT results (black dashed line). e) Brillouin Zone with relevant high symmetry points for cubic LiH.

2.2 RMSEs

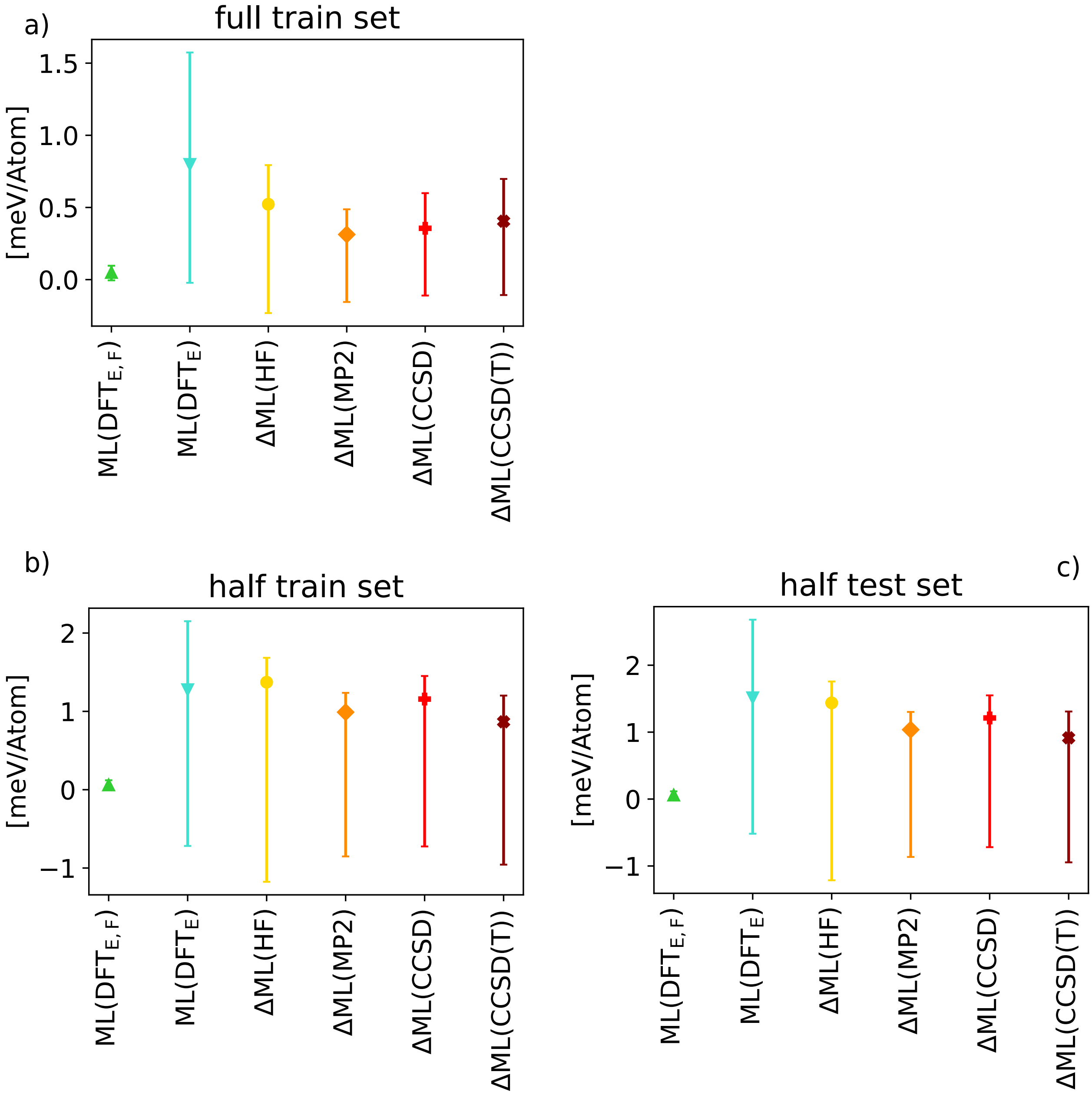

Fig. S.5 RMSEs for full train set (a), half train set (b) and half test set (c) for various MLFFs. ΔMLs always refer to errors from the combination of ML(DFT$_{E,F}$) and ΔML. Spreads in error represent the different seeds.

2.3 Seed Lines

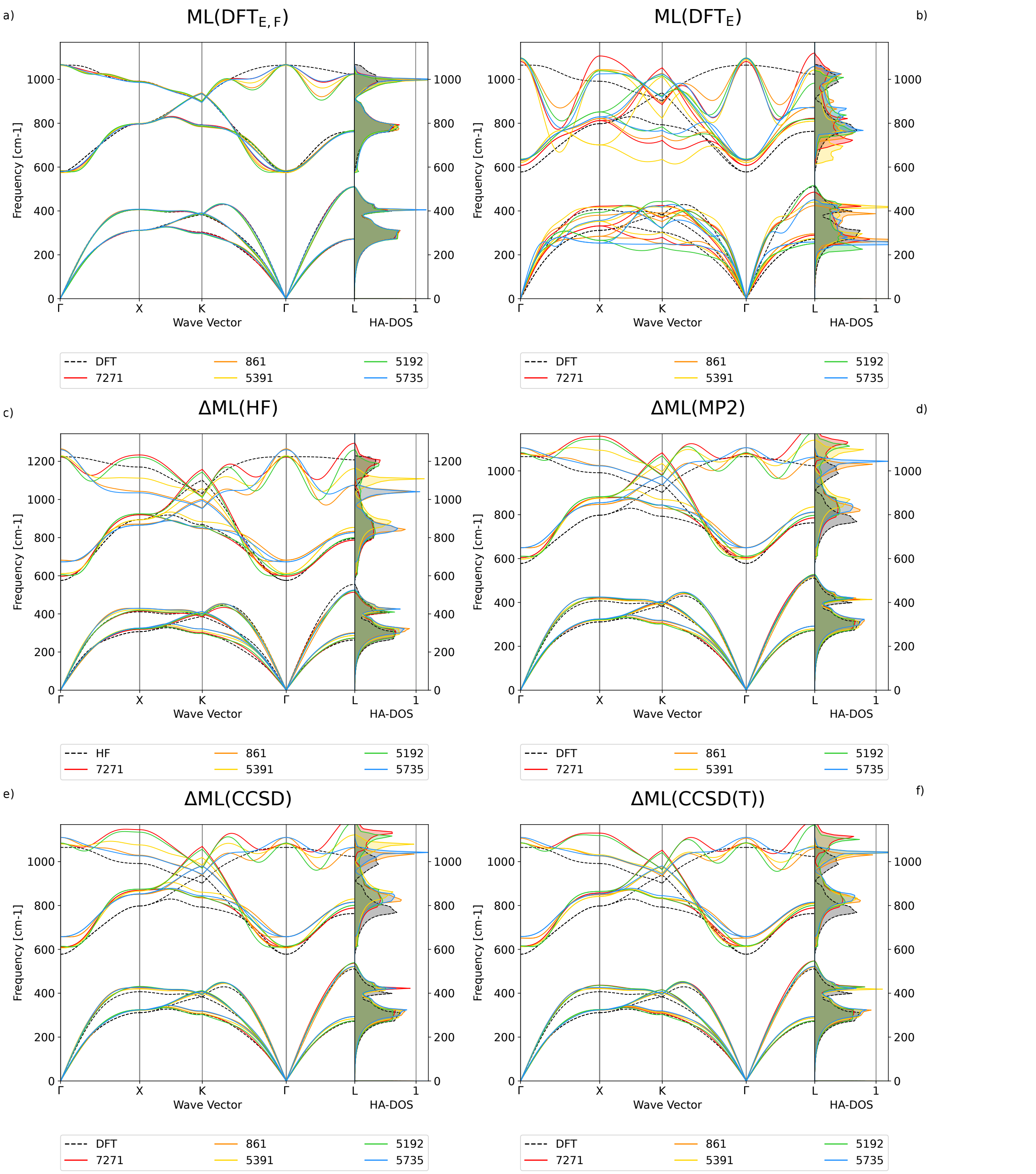

Fig. S.6 LiH Phonons showing individual lines for each seed (red, orange, yellow, green, blue lines) for ab initio DFT (black dashed line) and a) ML(DFT$_{E,F}$) b) ML(DFT$_{E}$) c) ΔML(HF) d) ΔML(MP2) e) ΔML(CCSD) f) ΔML(CCSD(T)).

3 Lithium Hydride - QNEP

3.1 Half Sets

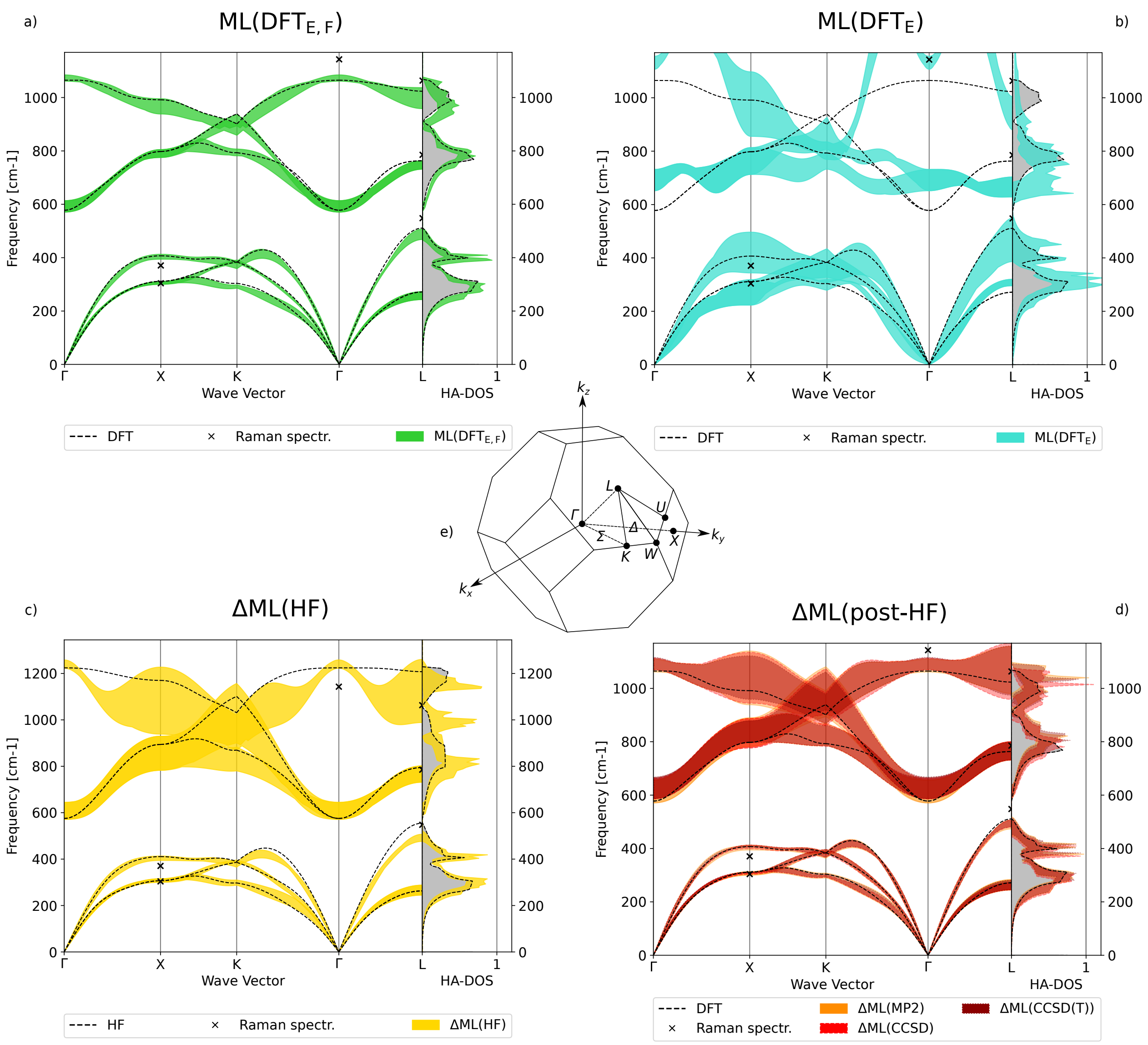

Fig. S.7 Phonon dispersions and DOS comparisons for LiH using 998 training points for ML($DFT_{E,F}$) and 98 for the rest; Raman spectroscopy data (black x) from [4]. a) ML($DFT_{E,F}$) across various seeds (green area) vs. DFT results (black dashed line). b) ML(DFT_E) (trained only on E) across various seeds (cyan area) vs. DFT results (black dashed line). c) ΔML(HF) across various seeds (yellow area) vs. HF results (black dashed line). d) Different ΔML(WFT) results across various seeds (gold, red dashed, purple dotted areas) vs. DFT results (black dashed line). e) Brillouin Zone with relevant high symmetry points for cubic LiH.

3.2 Seed Lines

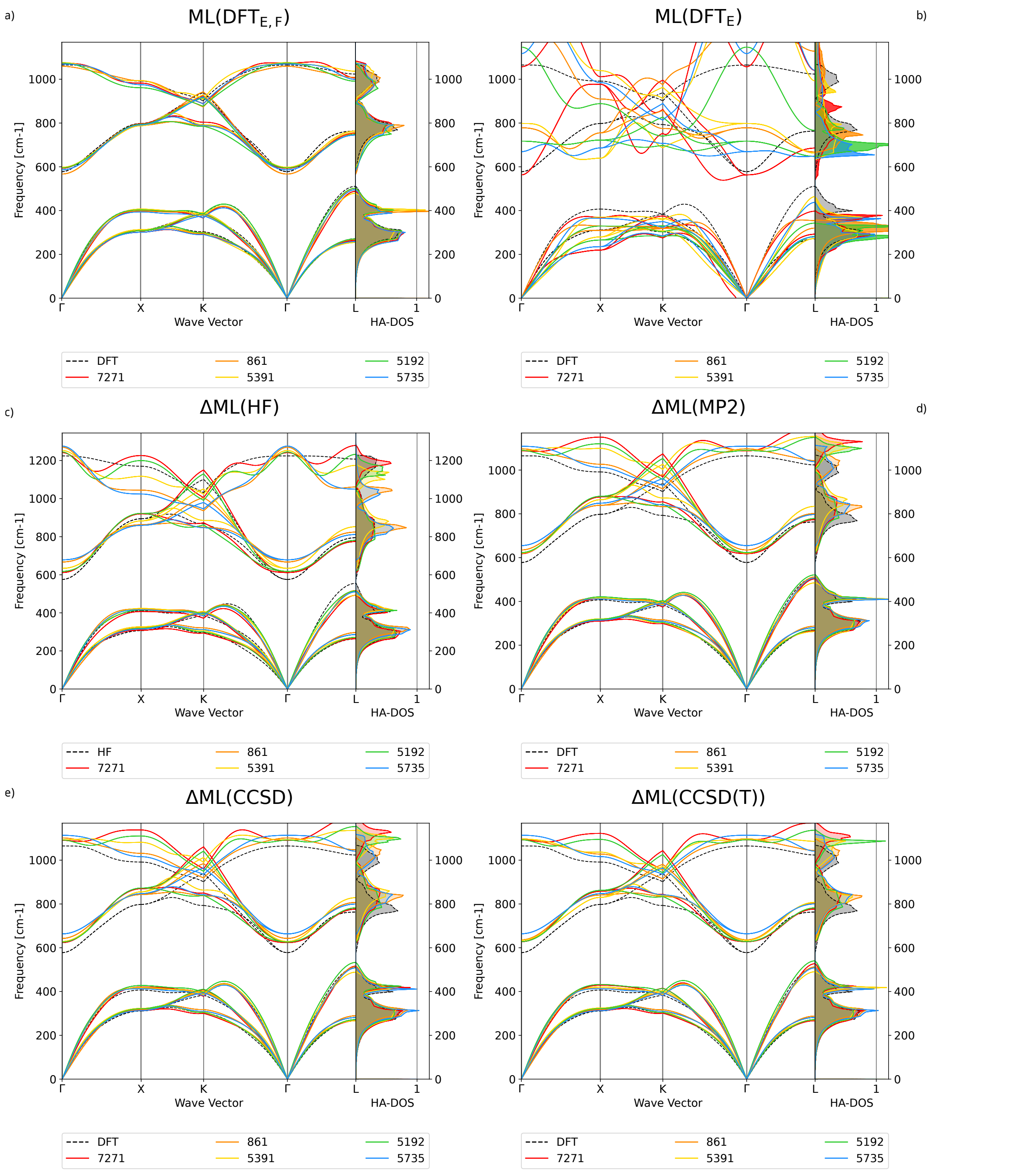

Fig. S.8 LiH Phonons showing individual lines for each seed (red, orange, yellow, green, blue lines) for ab initio DFT (black dashed line) and a) ML($DFT_{E,F}$) b) ML(DFT_{E}) c) ΔML(HF) d) ΔML(MP2) e) ΔML(CCSD) f) ΔML(CCSD(T)).

References

[1] Warren, J.L., Yarnell, J.L., Dolling, G., Cowley, R.A.: Lattice dynamics of diamond. Phys. Rev. **158**, 805–808 (1967) https://doi.org/10.1103/PhysRev.158.805

[2] Occelli, F., Loubeyre, P., LeToullec, R.: Occelli, f., loubeyre, p. & letoullec, r. properties of diamond under hydrostatic pressures up to 140 gpa. nature mater. 2, 151-153. Nature materials **2**, 151–4 (2003) https://doi.org/10.1038/nmat831

[3] Maezono, R., Ma, A., Towler, M.D., Needs, R.J.: Equation of state and raman frequency of diamond from quantum monte carlo simulations. Phys. Rev. Lett. **98**, 025701 (2007) https://doi.org/10.1103/PhysRevLett.98.025701

[4] Roma, G., Bertoni, C.M., Baroni, S.: The phonon spectra of lih and lid from density-functional perturbation theory. Solid State Communications **98**(3), 203–207 (1996) https://doi.org/10.1016/0038-1098(96)00067-1
